# Multimodality cardiac imaging of a left ventricular thrombus: a case report

**DOI:** 10.1186/s13104-015-1024-0

**Published:** 2015-02-28

**Authors:** Vineet Goyal, Hilary Bews, Nasir Shaikh, Farrukh Hussain, Davinder S Jassal

**Affiliations:** Institute of Cardiovascular Sciences, St. Boniface Research Centre, University of Manitoba, Winnipeg, Manitoba Canada; Section of Cardiology, Department of Internal Medicine, Faculty of Medicine, University of Manitoba, Winnipeg, Manitoba Canada; Department of Radiology, Faculty of Medicine, University of Manitoba, Winnipeg, Manitoba Canada; Section of Cardiology, Department of Internal Medicine, College of Medicine, Faculty of Health Sciences, University of Manitoba, Rm Y3531, Bergen Cardiac Care Centre, St. Boniface General Hospital, 409 Tache Avenue, Winnipeg, Manitoba R2H 2A6 Canada

**Keywords:** Ventriculography, Cardiac MRI, Transthoracic echocardiography, Left ventricular thrombus

## Abstract

**Background:**

Left ventricular thrombus (LVT) formation occasionally complicates patient recovery post myocardial infarction, conveying a significant risk of systemic embolism. Accordingly, thrombus detection and subsequent anticoagulation is imperative in order to minimize patient morbidity and mortality. Transthoracic echocardiography (TTE) is the imaging modality most widely used to screen for thrombus formation despite its suboptimal sensitivity and specificity.

**Case presentation:**

This report describes the discordant imaging findings of a LVT in a 56 year old Caucasian male with an anterior ST elevation myocardial infarction. Left ventriculography revealed a filling defect, suggestive of a potential left ventricular (LV) thrombus, which could not be confirmed by TTE. Cardiac magnetic resonance imaging (MRI) demonstrated evidence of a full thickness scar involving the mid to distal anterior wall and apical regions, with confirmation of a small LV apical thrombus.

**Conclusions:**

This case illustrates the limitations of TTE when used as a tool to screen for thrombus formation. It highlights the importance of multimodality cardiac imaging for the detection of post myocardial infarction (MI) complications, in the context of a high clinical suspicion.

## Background

Left ventricular thrombus (LVT) formation constitutes a significant concern for patients post myocardial infarction (MI). Approximately 4% of ST elevation myocardial infarction (STEMI) patients treated aggressively with primary percutaneous intervention (PCI) therapy demonstrate evidence of a LVT by contrast echocardiography [1]. The majority (75%) of LV thrombi are apical in location, reflecting common patterns of injury and blood stasis [2,3]. MI plays a role in the pathogenesis of a LVT by contributing to Virchow’s Triad of predisposing factors including: i) endothelial injury from ischemic insult; ii) hypercoagulability secondary to an increase in procoagulant factors; and iii) stasis from akinetic necrotic myocardium, especially at the apex [4]. Certain cardiac characteristics, representative of transmural infarcts and encompassing components of Virchow’s Triad, have emerged as predictors of LVT formation. These include a reduced ejection fraction (EF), anteriorly located infarct, regional wall motion abnormalities, and multiple aneurysms [1,3,5].

The significance of LV thrombi stems not only from its epidemiology but also its associated morbidity and mortality; a LVT often accompanies larger infarcts and conveys the risk of systemic embolism [1,3]. Thus, treatment of a LVT is aggressive, involving anticoagulation with warfarin for at least three months [6,7]. Novel anticoagulants, such as dabigatran, have yet to be approved for LVT anticoagulation, despite extensive use for stroke prevention in atrial fibrillation [7]. The convenience of transthoracic echocardiography (TTE) has led to its widespread use in the detection of LV thrombi, despite certain limitations. Its efficacy however, is improved with the concomitant use of contrast or other imaging modalities.

## Case presentation

**Figure 1:**
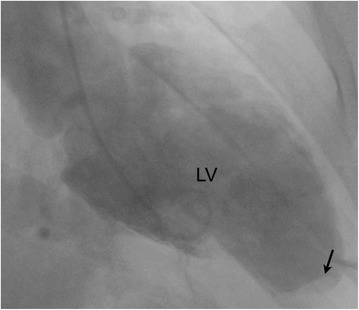
**Left ventriculography in a right anterior oblique view demonstrating a filling defect within the left ventricular apex suggestive of an apical thrombus.**

**Figure 2:**
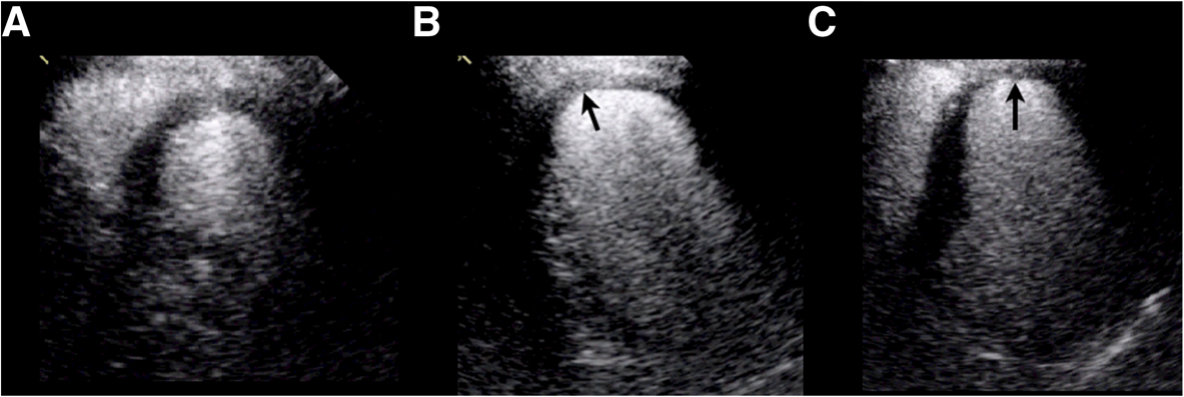
**Parasternal short axis view (A), apical 2 chamber view (B), and apical 4 chamber view (C) on transthoracic echocardiography with contrast demonstrating no evidence of an apical thrombus.**

**Figure 3:**
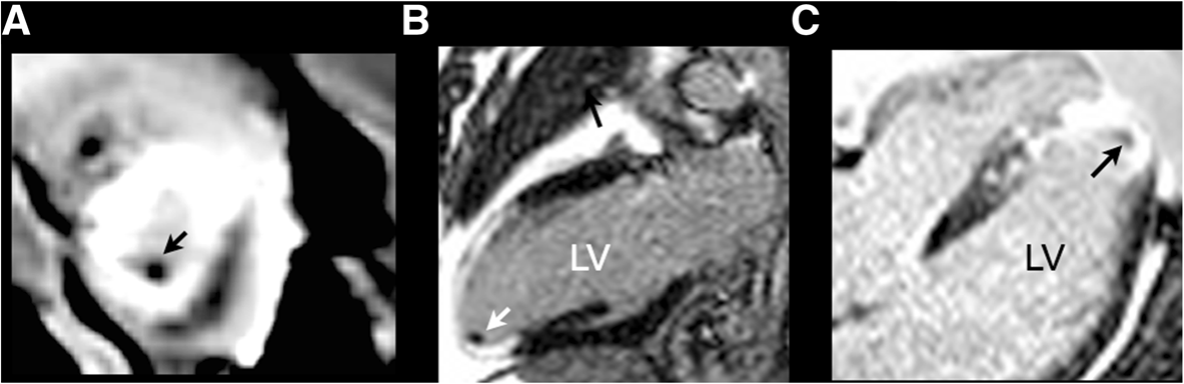
**Delayed enhancement cardiac magnetic resonance imaging sequence in the short axis view (A), apical 2 chamber view (B), and apical 4 chamber view (C) confirming full thickness scar involving the mid to distal anterior wall and apical regions, with delineation of a small left ventricular apical thrombus measuring 10 x 5 mm in dimension.**

A 56 year old Caucasian male with an anterior STEMI underwent PCI of the left anterior descending artery with a drug eluting stent. Left ventriculography revealed a filling defect within the inferoapical region suggestive of a potential LV thrombus (Figure 1). Subsequent TTE with contrast confirmed mild LV systolic dysfunction with an EF of 40-45% with akinesis of the apex. However, a LV apical thrombus could not be confirmed on contrast TTE (Figure 2). Due to this discrepancy in findings, cardiac MRI (CMR) was performed which demonstrated evidence of full thickness scar involving the mid to distal anterior wall and apical regions, with confirmation of a small LV apical thrombus measuring 10 x 5 mm in dimension (Figure 3). Multimodality cardiac imaging may be required in the clinical detection of post MI complications, including an apical thrombus.

## Discussion

This case illustrates the importance of multimodality imaging in the clinical detection of a LVT when a high degree of suspicion exists. TTE alone was unable to adequately visualize the source of the filling defect demonstrated by left ventriculography. The use of CMR was necessary in this report to confirm the presence of a thrombus, serving to highlight its superior sensitivity and specificity, as well as the limitations of other imaging modalities in thrombus detection.

Left ventriculography is a useful imaging modality to screen for LVT in patients undergoing cardiac catheterization post-MI, as was seen in this case. Thrombi appear as discrete filling defects in areas of akinesia or dyskinesia [8]. Ventriculography, however is limited by a low sensitivity (31%) and specificity (75%), which may directly result from a number of factors: failure of contrast to mix in dyskinetic areas, obscuration of filling defects by overlying material, and/or difficulty differentiating thrombi from normal cardiac architecture, such as trabeculations [8]. As a result, abnormal vetriculographic findings are verified by TTE.

TTE remains the imaging modality most widely used to screen for LV thrombi, as it is cost effective, accessible, and non-invasive. A LVT appears as an echo-dense mass distinct from the LV wall, but adjacent to an area of abnormal wall motion [5]. Despite the widespread use of TTE, diagnostic performance is suboptimal with a sensitivity of only 33% and specificity of 91% [9]. TTE can be technically challenging as a result of an indistinguishable myocardial-thrombus interface, foreshortening of the LV apex or poor visualization of small protuberant thrombi or mural thrombi of any size [3]. In fact, TTE can only detect around 10% of thrombi less than 1 cm in size, which may explain why the LVT was not detected by TTE in this clinical case [3]. Contrast echocardiography has been shown to significantly improve detection of a LVT (sensitivity and specificity are 61% and 99% respectively), enhancing endocardial border definition and overall image quality [2]. Thus, contrast echocardiography is recommended for consideration when standard imaging proves inconclusive, which may be in as many as half of imaging studies [10].

In contrast to TTE, which identifies a LVT based on anatomic appearance, delayed enhancement CMR (DE-CMR) relies on tissue characterization. The avascular LVT is viewed as an absence of contrast uptake adjacent to hyperenhanced scarred myocardium [3]. DE-CMR is superior to other imaging modalities in regards to LVT detection, with a sensitivity of 88% ± 9% and specificity of 99% ± 2% [3]. Furthermore, DE-CMR is better suited for detecting small thrombi; sensitivity only begins to decrease when imaging masses of less than 1 cm in size [3]. Therefore, when other imaging modalities demonstrate discordant findings, the use of CMR is warranted in order to rule out potentially fatal complications for patients post MI.

## Conclusion

The current case illustrates the difficulty associated with LVT detection, as imaging modalities used to screen patients post MI are imperfect. When clinical suspicion is high and imaging results discordant, a third modality should be sought.

## Consent

Written informed consent was obtained from the patient for publication of this Case report and any accompanying images. A copy of the written consent is available for review by the Editor-in-Chief of this journal.

## Abbreviations

(CMR): Cardiac magnetic resonance imaging
(DE-CMR): Delayed enhancement CMR
(EF): Ejection fraction
(LV): Left ventricle
(LVT): Left ventricular thrombus
(MI): Myocardial infarction
(PCI): Primary percutaneous intervention
(STEMI): ST elevation myocardial infarction
(TTE): Transthoracic echocardiography
